# Service Evaluation: Patient Engagement With Online Group Psychotherapy During the COVID-19 Pandemic in West of Scotland

**DOI:** 10.1192/bjo.2022.419

**Published:** 2022-06-20

**Authors:** Christos Vatalis, Lorraine McGuinness, Elizabeth Ogston

**Affiliations:** North Lanarkshire Psychotherapy Service, Coatbridge, United Kingdom

## Abstract

**Aims:**

Online group therapy has gained popularity during the COVID-19 pandemic as an alternative to face-to-face group therapy. This service evaluation is aimed at assessing how this paradigm shift is received by the patients and how the quality of the provided services is assured and potentially improved.

**Methods:**

1. We have retrospectively compared the attendance records from all psychotherapy groups in North Lanarkshire psychotherapy department between two distinct time periods: from November 2018 to January 2019 when only face-to-face groups were available (5 groups, 29 patients) and from November 2021 to January 2022 when only online groups were available (4 groups, 21 patients).

2. In order to prospectively assess patient's views regarding online group therapy, Client Satisfaction Questionnaires (CSQ-4, quantitative and qualitative) were distributed to January 2022 groups only.

**Results:**

1. The attendance rate was 5% lower in the face-to-face groups (76% vs 81%) while the non-attendance rate was lower by 1% in the online groups (9% vs 8%). The cancellation rate was also smaller by 5% in the online groups (14% to 9%).

2. In regard to the service satisfaction rates in the online groups, 57% of patients who responded, answered that “most of their needs have been met by the online services’’, 71% answered “services helped with their problems somewhat’’, 71% answered “they think they would come back to the program again and 66% answered” they are mostly satisfied with the services received.“The predominant positive aspects of the services according to free text comments were” “communication, understanding, sense of community’’ and the negative aspects that need improvement: “return to face-to-face (71% of answers), need personal interaction’’.*

* We have extended the deadline for the acceptance of the responses to 20th of February 2022 due to mailing systems being slowed down by the pandemic. (7 out of 21 questionnaires have been returned)

**Conclusion:**

Overall the attendance rates between the online and face-to-face group therapy exhibit minor differences. Concerning the patient satisfaction rates they reveal that the majority of patients who receive group therapy online are above-average satisfied with the services, feel that the online therapy provides a sense community but would prefer to return to face-to-face therapy.

Further data and studies will be needed to reach more robust conclusions.

